# Wavelength-Dependent Metal-Enhanced Fluorescence Biosensors via Resonance Energy Transfer Modulation

**DOI:** 10.3390/bios13030376

**Published:** 2023-03-13

**Authors:** Seungah Lee, Seong Ho Kang

**Affiliations:** Department of Applied Chemistry, Institute of Natural Sciences, Kyung Hee University, Yongin-si 17104, Gyeonggi-do, Republic of Korea

**Keywords:** metal-enhanced fluorescence, resonance energy transfer, carbon nanodots, noble metals, upconversion nanoparticles, biosensor, ultra-sensitivity

## Abstract

Fluorescence can be enhanced or quenched depending on the distance between the surface of a metal nanoparticle and the fluorophore molecule. Fluorescence enhancement by nearby metal particles is called metal-enhanced fluorescence (MEF). MEF shows promising potential in the field of fluorescence-based biological sensing. MEF-based biosensor systems generally fall into two platform categories: (1) a two/three-dimensional scaffold, or (2) a colloidal suspension. This review briefly summarizes the application studies using wavelength-dependent carbon dots (UV-VIS), noble metals (VIS), and upconversion nanoparticles (NIR to VIS), representative nanomaterials that contribute to the enhancement of fluorescence through the resonance energy transfer modulation and then presents a perspective on this topic.

## 1. Introduction

Fluorescence is one of the dominant detection/sensing methodologies for the biological and chemical sciences [1]. Fluorescence can act as an excellent sensing probe because fluorescent materials can change emission intensity or wavelengths as they interact with ligands or other chemical agents. In fluorescence-based optical biosensors, fluorescence probes act as effective converters for transmitting fluorescence signals in biometric events collected using various detectors [2]. Despite the attractive features of fluorescence detection, fluorescence signals may not be sufficiently intense when analyte concentrations are low. Thus, new strategies are needed to improve signal collection and to achieve high sensitivity in appropriate dynamic ranges [3].

In 1948, Förster resonance energy transfer (FRET, IUPAC nomenclature) was theoretically established by Theodore Förster [4]. Förster discovered that energy transfer through dipole coupling between molecules depends primarily on two factors: spectral overlap and intermolecular distance, and discovered the *R***^−^**^6^ distance dependence law for the rate of resonance energy transfer (RET) over short distances [5]. Until now, RET technology has been widely applied to study various intermolecular interactions (fluorescence RET [6], bioluminescence RET [7], chemiluminescence RET [8], nanometal surface energy transfer [9], and plasmon RET [10]). In 2007, Lee’s group showed that the plasmon resonance energy of NPs could be transferred to nearby cytochrome-*c*. This interaction was called plasmonic resonance energy transfer (PRET) [10]. Gao et al. introduced nanometal surface energy transfer and PRET techniques using plasmonic nanoparticles (NPs) [11]. The physical mechanisms, efficiency measures, and principles of RET have already been summarized in many papers [11,12,13,14]. Metal NPs are associated with luminescence enhancement and quenching phenomena, which are energy transfer pathways. Typically, the signal is quenched when the fluorophores-to-metal separation distance is within about 5 nm and enhanced within the optimal distances typically between 5 and 90 nm [2]. We called the latter metal-enhanced fluorescence (MEF).

With the rapid development of nanotechnology, the combination of fluorescent probes and nanometallic materials has presented an opportunity to further upgrade the reliability and sensitivity of biosensor systems. As a representative example, MEF-based biosensors have the potential to improve current fluorescence-based techniques, such as single-molecule sensing, bioimaging, DNA sequencing, or disease identification [15,16,17]. MEF refers to a system in which the electromagnetic field in the area adjacent to metal NPs enhances the optical signal of nanomolecules or NPs close to the metal surface. This interesting phenomenon effectively combines metal nanostructures with fluorophore molecules used for targeted molecular detection, leading to higher sensitivity and lower detection limits compared to conventional optical biosensors [2,18,19]. Such MEF-based methods to enhance fluorescence detection sensitivity for detecting molecules at ultra-low concentrations are already being applied to biosensor systems [12].

In order to achieve the best enhancement factor, great efforts have been made to design various plasmonic nanomaterial structures that would maximize the radiative emission of the fluorophores and control the distance between the nanomaterial and the fluorophore molecule [12,20,21,22]. Previously, Jeong et al. reviewed MEF’s general approaches and recent developments for biosensors. They reported that the MEF phenomenon can occur in all fluorescent nanomaterials as well as organic fluorophores [2]. In recent years, there has been significant research interest in the development and application of metal nanomaterials for fluorescence enhancement. Among them, wavelength-dependent carbon nanodots (CNDs), noble metals, and upconversion NPs (UCNPs) have been extensively studied due to their ability to modulate RET depending on the spectral range. The optical properties of these metal nanomaterials can be controlled by adjusting their size, surface chemistry, and excitation wavelength, making them highly tunable for specific applications. For instance, wavelength-dependent CNDs can enhance fluorescence in the UV-VIS range, while noble metals such as gold and silver can enhance fluorescence in the VIS range through surface plasmon resonance. UCNPs can convert NIR into VIS light, which is ideal for deep-tissue imaging. These metal nanomaterials have great potential for bioimaging, sensing, and other fields.

This mini-review briefly summarizes application studies using wavelength-dependent CNDs (UV-VIS), noble metals (VIS), and UCNPs (NIR to VIS) that contribute to fluorescence enhancement through RET modulation among numerous metal nanomaterials by classifying a wide spectrum into three ranges. Furthermore, we focused on examples of various technologies designed to control plasmonic nanostructures (nanoarchitecture) that can be used to develop various sensor platforms and control the distance between fluorophores and metal NPs to realize the full potential of MEF technology.

## 2. Carbon Nanodots in the Ultraviolet-Visible Region

Carbon nanodots (CNDs), used as a “nanolight”, are emerging as an attractive fluorescent carbon material due to their strength, adjustability, photoluminescence and good optoelectronic properties [23,24,25]. Several chemical sensors have been developed to detect biomolecules [26] and metal ions [27,28] based on the quenching and recovery of CND fluorescence. In particular, the emission enhancement of CNDs is of great interest to the development of nanophotonics [29]. However, while plasmon-enhanced fluorescence phenomena for quantum dots (QDs) and fluorophore molecules have been well investigated, low quantum yield (<5%) for CNDs limit their application in biological studies [30,31,32,33,34,35]. Therefore, the important of research on CND-based MEF chemical sensors and light emission improvement is required for biological applications.

**Figure 1:**
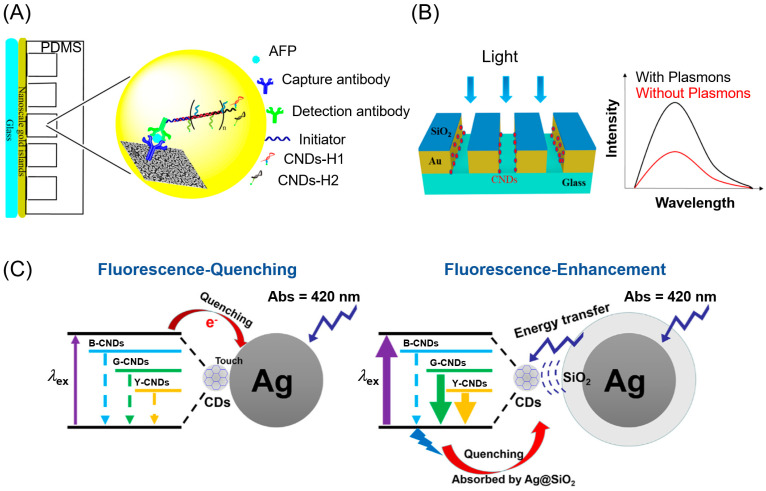
(**A**) Schematic illustration for the detection of AFP with Immuno-HCR and MEF of CNDs [36]. (**B**) Illustration of self-assembled monolayer formation and CND immobilization on a gold nanoslit surface [29]. (**C**) Electron transition process between CNDs and Ag or Ag@SiO_2_ NP for quenching (**left**) and enhancement (**right**) of fluorescence [39]. Reprinted with permission from [29,36,39].

Xu et al. constructed a dual amplification fluorescence sensor based on an immuno-hybridization chain reaction (immuno-HCR) and MEF of CNDs for the detection of *α*-fetoprotein (AFP) (Figure 1A). A capture antibody-coated Au film slide captured the target molecule, AFP, and a detection antibody-conjugated oligonucleotide initiator was introduced to bind the AFP target. The introduction of a CND-tagged DNA hairpin allowed the DNA hairpin and oligonucleotide initiators to trigger complementary HCRs. Using this gold film and CNDs, the radiative decay rate was greatly improved, increasing the quantum yield and enhancing the fluorescence emission of the CNDs. They achieved a detection limit of 1.35 fM (94.3 fg/mL), as well as a 0.992 coefficient of correlation in the concentration range from 7.14 fM to 71.43 pM (0.0005 to 5 ng/mL). Fluorescence emission of plasmon slides was improved up to nearly seven-fold over non-plasmon slides by the effect of MEF at the same concentrations of AFP [36]. This result is more sensitive than that reported by Wu et al. in 2015, who measured the detection of human AFP using CNDs bound to labeled anti-AFP by cross-linking with glutaraldehyde through a high-throughput well plate-based immunosorbent assay [25]. Bagra et al. designed plasmonic nanoslits to immobilize CNDs and observe the fluorescence enhancement of CNDs. Maximum fluorescence and surface-reflected light intensity enhancement were obtained with a 100 nm nanoslit width, suggesting that plasmon light trapping was responsible for an increased electromagnetic field and plasmon-induced RET (Figure 1B) [29].

Fluorescence enhancements have been demonstrated in the blue-green range by binding CNDs to silver nanoparticles (AgNPs) [37,38,39]. Figure 1C illustrates an electron transition and energy transfer process between a CND and AgNP or AgNPs coated with a SiO_2_ layer (Ag@SiO_2_ NP) using a fluorescence quenching or enhancement mechanism, respectively [39]. Plasmonic enhancement was more pronounced when CNDs were nano-hybridized to silver rather than gold due to low intrinsic loss and superposition between the absorption spectra of metallic silver and CNDs [38]. CNDs can be operated like nanoantenna through two-way energy exchange with nearby chromophores. Recently, Sciortino et al. exploited the overlap between the surface plasmon resonance of gold and the electronic transitions of carbon dots to increase the fluorescence by a factor of 5 in the normally very weak orange region [40]. In their study, they elucidated that the underlying mechanism of enhancement was through coherent RET occurring in less than 70 fs from metal nanoantennas to CNDs.

Deng et al. synthesized visible-ultraviolet upconversion CNDs to enhance the photocatalytic activity of titanium dioxide [41]. CNDs were synthesized by a hydrothermal method using *L*-glutamic acid (*L*-Glu) and *m*-phenylenediamine (MPD), and they were combined with commercial nano-TiO_2_ to prepare a CNDs/TiO_2_ complex, proving that CNDs can convert about 600 nm visible light to 350 nm ultraviolet light. Their successful work has provided an opportunity to further expand the application range of upconversion materials and the utilization efficiency of light sources.

CNDs have indeed been extensively studied due to their unique optical properties, biocompatibility, and potential for various applications including bioimaging, sensing, and drug delivery. In addition, the synthesis of biomass-derived CNDs is an excellent example of the efforts to make CNDs more environmentally friendly [42]. However, the excitation wavelengths of CNDs are limited to their absorption bands, which may be limited in some applications. Furthermore, with regard to its use as a detection probe, several strategies have been proposed to reduce background fluorescence, such as surface immobilization or surface functionalization, but it still can lead to false-positive results due to the high signal-to-noise ratio by autofluorescence in CND.

## 3. Noble Metals in the Visible Region

Noble metal nanostructures possess unique optical properties as well as good biocompatibility, satisfactory stability, and multiplex functionality. These unique advantages make noble metal nanostructures an ideal medium for developing biosensing and bioimaging methods when used as colloidal suspensions or 2D/3D substrates for MEF studies. Typically, MEF occurs when the fluorophore is positioned at an optimal distance (5–20 nm) from the noble metal [43,44,45], leading to improved quantum yield, reduced fluorescence lifetimes, and increased photostability.

**Figure 2:**
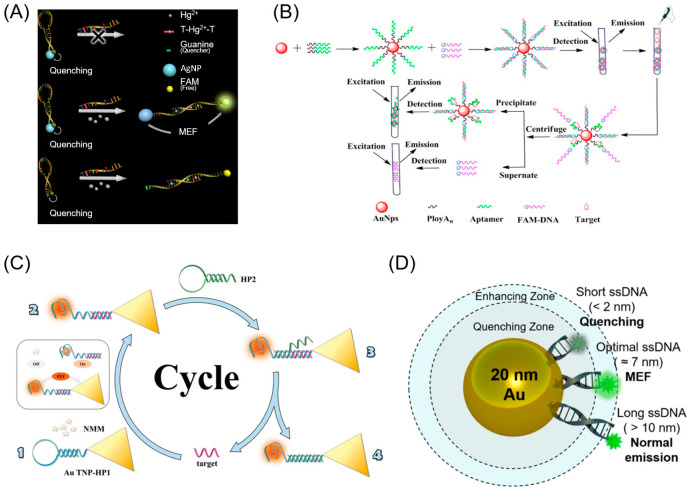
(**A**) Schematic illustration of the distance-dependent MEF sensing platform based on a molecular beacon [46]. (**B**) Schematic illustration of the preparation of MEF-based AuNPs@polyA_n_-aptamer@FAM-DNA nanostructure and target detection [48]. (**C**) Schematic illustration of target DNA fluorescence detection based on the CHA and plasma resonance fluorescence (PEF) effect [52]. (**D**) Schematic diagram of the MEF effect dependent on the DNA length of dsDNA between 20-AuNPs and FITC [53]. Reprinted with permission from [46,48,52,53].

Various techniques have been devised as fluorescent aptasensors by controlling the distance between the fluorophores and the noble NPs according to the MEF principle. Zhou et al. evaluated the fluorophore-metal distance effect and the AgNPs size effect on fluorescence enhancement. Fluorescently labeled molecular beacons were attached to AgNPs using recognition moieties. In addition to reducing the reagent blank signal, AgNPs provided an electric field superimposed on the incident light to increase the fluorescence intensity when the closed hairpin was opened (Figure 2A) [46]. When a 12 nm thick silica spacer shell was introduced between the gold nanoparticles (AuNPs) and gold nanoclusters (AuNCs), the fluorescence enhancement from AuNCs by Au@SiO_2_NPs was enhanced 3.72 times [47]. Jiang et al. hybridized polyA_n_-based special nucleic acid aptamers modified on the surface of AuNP with fluorescein amidite (FAM)-labeled single-strand oligonucleotides to enhance fluorescence signals by up to 2.7 times and detect adenosine triphosphate (ATP) with a detection limit of 0.2 nM (Figure 2B) [48]. By placing QDs in the vicinity of metal NPs, the intrinsic excitation and relaxation processes of QDs were significantly modified, resulting in enhanced fluorescence intensity. Recently, Kim et al. used heavy metal-free InP@ZnS core-shell QDs instead of toxic Cd-based QDs and chose Au@Ag@SiO_2_ core-shell NPs for the localized surface plasmon resonance (LSPR) effect. Systematic control over the thickness of the SiO_2_ shell enabled enhancements in QD fluorescence in poly(lauryl methacrylate) (PLMA) composite films, up to 12.9 times its original intensity [49]. Lotfi et al. applied the optimized fabricated silver dendritic nanostructure as a platform for the development of a MEF-based aptasensor for thrombin detection. After immobilizing a thiolated 29-mer thrombin-binding aptamer (TBA_29_(12T)–SH) as a capture aptamer on the surface of the silver dendritic nanostructure, thrombin was sandwiched between the capture aptamer and the Cy5-labeled 15-mer thrombin aptamer (TBA15-Cy5). The aptasensor they developed could detect thrombin at a level of 32 pM [50]. Trotsiuk et al. performed a systematic experimental study of QD fluorescence enhancement near the surface of electrostatically deposited gold nanorods (GNRs) of polyelectrolytes. They found that the tendency of the experimental fluorescence intensity enhancement factor decreased with QD concentration, obeying the a + b*/x* dependence law. The maximum enhancement factor (up to 11 times) was observed in a complex with a two polyelectrolyte layer spacer between a GNR and QDs, and a QD/GNR ratio = 2.5 [51]. Recently, Shi et al. developed a highly sensitive DNA sensor for detecting HIV DNA fragments based on the signal amplification of plasma resonance fluorescence enhancements using a catalytic hairpin assembly (CHA) and an *N*-methyl mesoporphyrin IX (NMM)/G-quadruplexes system (Figure 2C). The sensor included a 5′ terminal connected to a triangular gold nanoplate, a 3′ terminal rich in guanine hairpin probes (HP1) and a hairpin probe (HP2) complementary to the partial subsequence of HP1. The plasmon resonance absorption peak of the triangular gold nanoplates overlaps the emission spectrum of the NMM fluorophore, and the fluorescence coupling between the fluorophore and the NP was strong, thus producing a fluorescence enhancement effect. The single-molecule counting method combined with the multiplex signal amplification strategy sufficiently amplified the signal to detect the HIV DNA fragment [52]. Choi et al. developed a CRISPR-Cas12a-based nucleic acid amplification-free fluorescent biosensor to detect cell-free DNA by MEF using DNA-functionalized 20 nm AuNPs. They confirmed the MEF effect according to the DNA length of dsDNA between AuNP and FITC (Figure 2D). The fluorescence signal intensity of the AuNP-dsDNA-FITC complex was maximally enhanced at the optimal distance of 7 nm, and the breast cancer biomarker (BRCA-1) was measured to a femtomolar limit of detection [53]. These structures successfully provided rigid separation distances between metal NPs and fluorophores, thereby converting fluorescence quenching into fluorescence enhancements.

**Figure 3:**
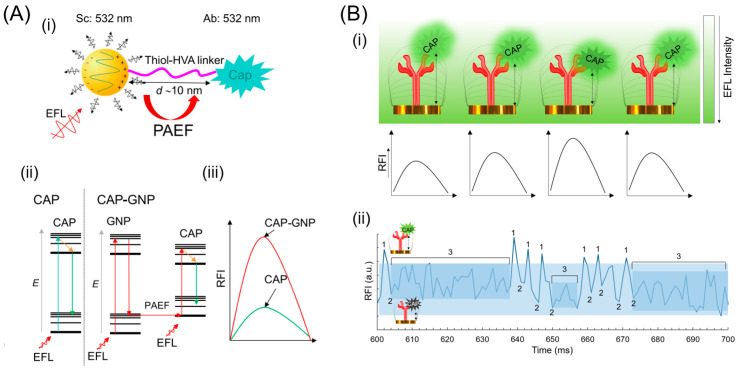
(**A**) (i) Conjugation of CAP with gold nanoparticle (GNP) via a thiol-antihomovanillic acid (HVA) linker. (ii) Schematic diagram of CAP fluorescence emission and CAP-GNP fluorescence enhancement emission. (iii) Schematic diagram of the CAP fluorescence enhancement after GNP conjugation [54]. (**B**) (i) Fluorophore fluctuation in an evanescent field layer after conjugation of GNI with CAP. (ii) Fluctuation-based observation of fluorophore position with regard to proximity of GNI. Numbers 1, 2, and 3 imply fluorescence enhancement, quenching, and equilibrium with regard to the distance between CAP and GNI [55]. Reprinted with permission from [54,55].

Chakkarapani et al. reported a simple, reliable, and highly sensitive endogenous fluorescence enhancement technique based on a single AuNP that can be used to detect endogenous fluorescent materials. They detected trace capsaicinoid (CAP) at 18 zM via self-endogenous fluorescence enhancement with plasmonic single NPs. Here, the inelastic scattering of plasmons was used as an electromagnetic field enhancer for the highly sensitive detection of ultra-trace amounts of endogenous phosphor. They named the method single nanoparticle plasmon-amplified endogenous fluorescence nanospectroscopic sensing (Figure 3A) [54]. In their recent work, they obtained cross-correlation quantification of CAP based on endogenous single-molecule fluorescence enhancement and quenching interfaces via correlated fluorescence fluctuation optical spectroscopy on plasmonic arrays of gold nanoislands (GNI). The endogenous fluorescence of CAP was dependent on the fluorophore–GNI distance and varied in enhancement, quenching, and equilibrium lifetimes (Figure 3B) [55].

**Figure 4:**
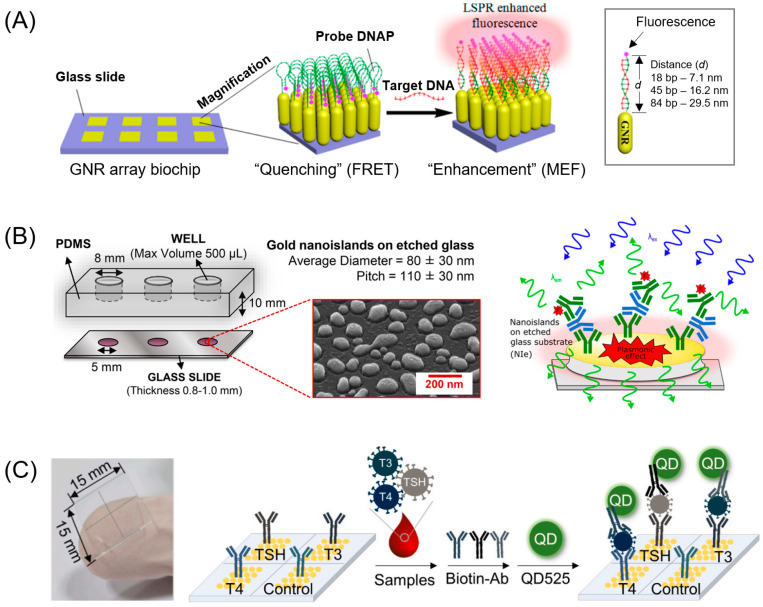
(**A**) Practical use of the ordered GNR array chip for DNA detection based on surface plasmon-enhanced fluorescence upon hybridization with the target [57]. (**B**) Randomly arranged gold nanodisks on an etched glass substrate [59]. (**C**) Photograph and diagram of the single-molecule immunoreaction process for three thyroid hormones on a fourplex nanoimmunosensor chip [62]. Reprinted with permission from [57,59,62].

Plasmonic nanostructures have been used to develop various sensor platforms, and analytes can be immobilized on the surface of nanostructures. Signal enhancement depends on the size and shape of the nanostructures (nanoholes, nanoarrays, nano-stripes, etc.) [2]. These MEF-active substrates have been applied to develop biosensors with trace detection limits. In 2013, Gartia et al. [56] demonstrated a metal-coated nano-well array for ultrasensitive refractive index sensing fabricated by an inexpensive large-area nanoreplication technique. Mei and Tang [57] vertically aligned GNRs on a glass surface as a plasmonic substrate to detect a molecular beacon (Figure 4A). This dramatically enhanced the LSPR between adjacent GNRs compared to an ensemble of random GNRs. Inserting an intermediate spacer of appropriate thickness between the fluorophore and the surface of the GNR array resulted in plasmonic coupling enhancement of the fluorescence signal up to 30 nm away. Zhang et al. mass-fabricated Au nanohole arrays by nanoimprinting using a 4-inch nickel mold [58]. These Au nanohole arrays showed high fluorescence enhancement through simultaneous excitation of localized surface plasmon (LSP) and propagating surface plasmon (PSP) modes. Simultaneous excitation of LSP and PSP modes resulted in a detection limit of 140 fM for a prostate-specific antigen (PSA) biomarker. Dual mode detection offered a seven-fold better than when only LSP was resonantly excited on the same substrate. Miranda et al. developed a randomly arranged MEF-based immunosensor of AuNPs that was fabricated through a three-step process to regulate the optical properties of size, interparticle distance, and optimal gold nanostructures (Figure 4B). These AuNPs enhanced the fluorescence emission of three fluorescent dyes (Alexa Fluor 488, Alexa Fluor 546, and PE Cy7) approximately 150-fold on a randomly arranged MEF immunosensor to achieve a detection limit of 28.67 pM (4.3 ng/mL) for immunoglobulin detection [59]. Kang’s group arranged 300 nm gold nanodisks using electron-beam lithography and used them as sensors for the high-sensitivity detection of biomolecules [60]. They designed the analyte to be sandwiched between a capture antibody immobilized on a gold nanodisk and a detection antibody conjugated with QDs. Using this, biogenic amines (histamine (His), tyramine (Tyr), and putrescine (Put)) and thyroid hormone markers (thyroid-stimulating hormone (TSH), triiodothyronine (T3), and thyroxine (T4)) were quantified by multiple simultaneous analysis, showing aM and yM detection limits, respectively (Figure 4C) [61,62]. More recently, Lee et al. proposed a turn-on sensing format based on enhancement of the emission signal of a fluorophore molecule close to plasmonic gold nanodisks rather than the generally used turn-off format, based on competitive reactions, for small-molecule quantification [63].

Most fluorescent nanomaterials or fluorophores close to noble metals such as gold or silver can detect small amounts of target molecules through fluorescence enhancement. However, MEFs rely on surface plasmon resonance (SPR) of noble metals, which can be sensitive to environmental factors such as temperature, pH and salt concentration. This may limit the reproducibility of MEF experiments. In addition, as mentioned above, not only fluorescence enhancement but also fluorescence quenching may occur, so care must be taken when designing experiments.

## 4. Upconversion Nanoparticles from Near-Infrared to Visible Region

Upconversion nanoparticles (UCNPs), a newly established fluorescent probe type, are excited by near-infrared light and emit visible light [64,65,66]. Compared to organic fluorophores and QDs, it has the property of greatly reducing background autofluorescence, photobleaching, photodamage, and the phototoxicity of biological specimens [67,68,69]. However, quantum yields using UCNPs are relatively low (~1%) due to their small absorption cross sections (ability to absorb photons of a specific wavelength and polarization) [70,71]. Compared to their bulk counterparts, the concentration of dopant ions on the surface of UCNPs is relatively high and can be quenched by surface quenchers, reducing the fluorescence intensity [72,73]. For this reason, research to enhance the upconversion emission of UCNPs is needed. Methods for enhancing the upconversion emission can be divided into two categories: (1) Controlling the absorption cross-section and radiative emission rate by changing the synthesis conditions of UCNPs. (2) Increasing the local photon density of states. In particular, the local field can be improved more than 10^2^ times using a plasmonic nanostructure [74]. In upconversion nanocrystals (UCNCs) that are able to convert lower energy photons (typically NIR) into higher energy photons (usually visible), the excitation and emission wavelengths can be substantially different and usually do not have significant overlap [75,76]. Therefore, it is significant to investigate the interplay between the excitation- and emission-plasmonic resonance coupling, as well as the possibility to distinguish their effects on plasmonic enhancement [77]. In this section, among the various methods to enhance the fluorescence sensitivity of UCNPs, a method using a plasmonic fluorescence enhancement strategy is presented.

**Figure 5:**
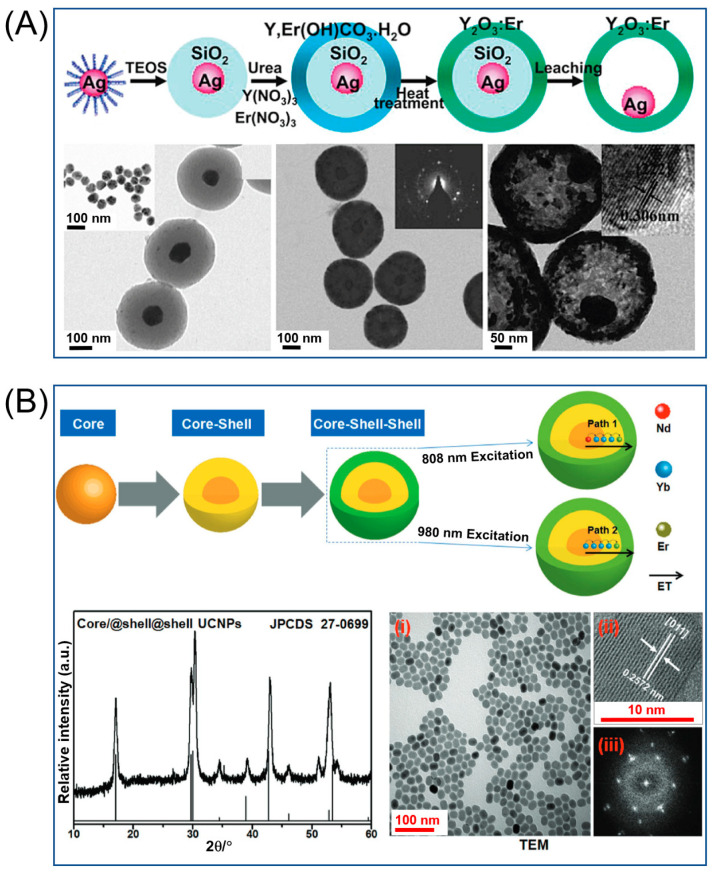
(**A**) Synthetic procedure for Ag@SiO_2_@Y_2_O_3_:Er nanostructures and TEM images of the resulting UCNPs [78]. (**B**) Design of a NaGdF_4_:Yb^3+^, Nd^3+^@NaGdF_4_:Yb^3+^, Er^3+^@NaGdF_4_ UCNC allowing for two different energy transfer paths [86]. Reprinted with permission from [78,86].

Dispersible plasmonic structures for biological applications can be deployed in bioimaging and therapeutic techniques, such as photodynamic therapy and drug delivery. Zhang et al. [78] easily fabricated a metal core-enhanced Er material based on a core/spacer/shell approach (Figure 5A). They switched the upconversion fluorescence enhancement and quenching phenomena by controlling the silica dielectric spacer thickness and metal core size. The results of this paper showed an optimal PL sensitivity when the space thickness was 30 nm, which was observed to be four times higher than that of the reference Y_2_O_3_:Er shell. In contrast, the use of thin SiO_2_ spacers resulted in PL quenching due to the proximity of the shell and Ag spheres and resulted in efficient nonradiative energy transfer from the luminescent system to the metal surface. Zhang et al. [77] reported a five-fold overall enhancement of upconversion emission in NaYF_4_:Yb/Er nanocrystals when coupled with gold island films. In contrast to isolated AuNPs [79,80,81,82], continuous gold films are characterized by a plasmonic resonance wavelength in the NIR region where the excitation wavelength (980 nm) for UCNPs is located [83,84,85]. Confocal fluorescence images showed that the upconversion emission increased more than five-fold when combined with the gold film. Kang et al. demonstrated that the excitation and release process of UCNPs can be simultaneously improved using these plasmonic double resonance nanostructures, thereby significantly increasing the UC luminescence (UCL) strength of UCNPs (Figure 5B) [86].

UCNP-based biosensors were used to detect proteins, nucleic acids, small molecules, and amino acids. In 2017, Du et al. demonstrated the usefulness of UCNPs as a bifunctional NP for in vitro cell imaging and latent fingerprint detection by synthesizing Ho^3+^-activated NaYbF_4_ UCNPs [87]. In 2019, Wu et al. [88] used an upconversion FRET sensor to detect arginine. They designed positively charged UCNPs as energy donors and negatively charged AgNP as energy acceptors. This method showed a good linear relationship between the fluorescence intensity and the concentration of arginine in 14.42–115.04 μM with a LOD of 2.9 μM. Kumar et al. [89] developed a UCNPs nanoplatform with mesoporous silica functionality to detect dopamine in real-time, wherein the LOD is as low as 0.63 nM. In 2020, Wang et al. [90] designed a new NIR probe based on FRET between UCNPs and Au nanocages for the detection of circulating tumor DNA (ctDNA). They determined excitation and emission at 980 nm and 800 nm, showing a linear range of 5–100 pM and a detection limit of 6.30 pM in serum. In 2021, Hu et al. [91] fabricated a new bilayer of poly(methylmethacrylate) opal photonic crystal (PMMA OPC) with a double photonic stop band, matching the excitation and emission fields of UCNP simultaneously to significantly enhance the upconversion fluorescence intensity. With 980 nm and 808 nm laser excitation, the PSA sensor showed a good linear relationship within the range of 2.94–294.12 pM (0.1–10 ng/mL) and LOD of 29.41 pM (0.01 ng/mL). For the detection of the cancer antigen 125 (CA125) biomarker, Zhang et al. [92] combined UCNPs modified with CA125 aptamer with CNDs as an energy acceptor through *π–π* stacking interaction. This CA125 sensor showed a linear range of 0.01–100 pM (0.01–100 U/mL) and a detection limit of 9 fM (9 × 10^−3^ U/mL). Recently, Liu et al. [93] reported a quantitative analysis of TSH by RET luminescence mechanism between the UCNPs donor and tetramethylrhodamine receptor. The resulting detection dynamic range and the limit of detection were 0.66–33.26 pM (0.1–5.0 mIU/L) and 0.43 pM (0.065 mIU/L).

**Figure 6:**
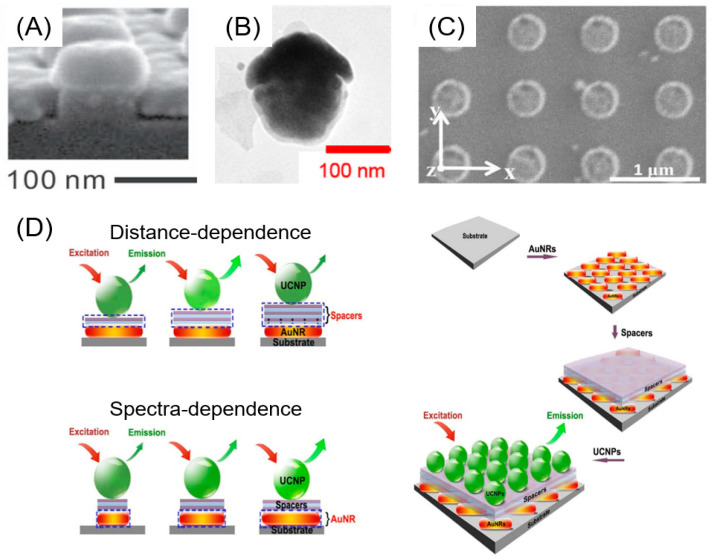
(**A**) SEM image of D2PA structure with different pillar heights on UC [88]. (**B**) TEM image of Y_2_O_3_:Yb, Er NPs with SiO_2_ spacer layer and Ag nanocap [89]. (**C**) SEM image of nanocylinder array with the diameter of 440 nm and height of 180 nm arranged in a square lattice [90]. (**D**) Distance- and spectra-dependence and fabrication process of the UCNPs and GNRs plasmon-enhanced fluorescence system [91]. Reprinted with permission from [89,90,91].

Applications of the non-dispersive plasmonic structures of UCNPs include multicolor flexible displays, fingerprints, solar energy harvesting, temperature sensing, and photocatalysis. The UCL enhancement of NaYF_4_:Yb^3+^/Er^3+^ co-doped nanocrystals was investigated using a disk-coupled dots-on-pillar antenna array (D2PA) with a 3D plasmonic nanoantenna architecture by Zhang et al. [94]. The D2PA structure was fabricated as a 3D nanocavity array, which consisted of Au nanodisks on the top of periodic dielectric pillars, an Au backplane at the feet of the pillars, and dense Au nanodots on the sidewalls (Figure 6A). Tuning the pillar height (*h* = 75 nm) could optimize the D2PA structure, resulting in a 310-fold uniform enhancement of UCL over a large area and an eight-fold reduction in luminescence decay time. Yamamoto et al. enhanced UCNP green emission by a factor of 23 and red emission by a factor of 43 through upconversion of 30 individual NPs composed of metal (Ag) nanocaps and rare-earth-doped UCNPs (Er- and Yb-doped Y_2_O_3_ NPs) [95]. UCNPs with silver nanocaps were formed by depositing a silver layer 10–50 nm thick on a quartz substrate spin-coated with UCNPs, and separating the substrate and UCNP using a polydimethylsiloxane (PDMS) stamp (Figure 6B). The intensity ratio of green and red luminescence was demonstrated to be dependent on the thickness of the Ag nanocaps, and the main cause of the upconversion enhancement is due to the enhancement of the radiative decay rates. Gao et al. placed an emitter layer in a diffractive array of Al nanocylinders to increase the absorption by rare earth ions. The array was designed to confine NIR in the emitter layer through the excitation of plasmon-photon hybrid modes, which are collective resonances of localized surface plasmons in nanocylinders through diffractive coupling, thus increasing the strength of the UCPL by up to seven times (Figure 6C) [96]. Plasmon nanostructures are known to efficiently enhance the fluorescence of surrounding fluorophores by acting as nanoantennae to focus the electric field into nano-spaces. Feng et al. reported a distance-dependent plasmon-enhanced fluorescence system by tuning the spacers between UCNPs and GNRs chosen as plasmon nanoantennae. A maximum upconversion enhancement up to 22.6-fold was achieved when the thickness of spacer polyelectrolyte multilayers was 8 nm and the LSPR absorption wavelength of GNRs overlap (~980 nm) with UCNPs excitation (Figure 6D) [97]. Eriksen et al. applied a rate equation model via a simulation-based approach to study the interaction between near-field enhancement and luminescence quenching over a range of geometries [98]. They used an excitation wavelength of 1523 nm for the simulation rather than the excitation wavelength of 980 nm considered in typical studies for plasmonic enhancement. Because of this, the enhancement factor values predicted by the simulations were lower than those reported in the literature. This suggests that the choice of excitation wavelength has a greater effect on increasing the efficiency of UC than the dependence on various plasmonic geometries.

Most recently, Meng et al. [99] realized single nanocrystal upconversion in a single plasmonic nanocavity mode by in situ controllable coupling and doping. An experimental platform was built to tune the nanocavity field and control the doping concentration of UCNCs. A single UCNC was placed between two GNR nanocavities so that all the activators of UCNC showed the same emission pattern and radiation efficiency. The outermost shell, as a protective layer, suppressed the energy transfer to surface defects, while the spacer layer mitigated the plasmonic quenching by coupling the UCL predominantly to the fundamental dipole mode. A nearly continuous evolution of the Purcell [100,101] enhancement for the same single nanocrystal was realized, which showed the general existence of UCL plasmonic enhancement saturation phenomena, as well as the doping- and coupling-dependent UCL enhancement factors up to 2.3 × 10^5^ [99].

UCNPs are actively pursued as anti-stock emitters for various applications, such as bioimaging, solar energy harvesting, catalysis, displays, anti-counterfeiting, sensing, and lasers [99]. To enhance UCL, researchers improved the internal material properties (composition, doping, and surface structure) of nanocrystals while simultaneously using plasmonic coupling to improve engineered external optical responses. However, despite impressive progress, upconversion brightness still fell short of the level required for commercial applications, and a systematic understanding of plasmon-enhanced upconversion remains a challenge.

## 5. Conclusions and Perspectives

In this review, we briefly reviewed recently reported papers on MEF-based biosensors designed to address the fundamental limitations of fluorescence-based detection, such as low quantum efficiency, photobleaching, and autofluorescence. Representative excitation wavelength-dependent nanomaterials (i.e., carbon dots, noble metals, upconversion nanoparticles) were introduced and verified to demonstrate great potential in terms of signal enhancement.

Despite these advantages, a selective nanomaterial molecular recognition element that can replace fluorophore molecules is still unavailable. The design and fabrication of nanostructures can potentially enhance fluorescence strength by three orders of magnitude compared to metal colloidal suspension, but it is expensive and time-consuming to manufacture rigid, uniform, and reproducible metal substrates. Thus, efficient nanostructure fabrication remains a task for current scientists in the manufacturing of smart nanomaterials and nanostructured surfaces.

In addition, despite many efforts to devise practical MEF biosensors, there are still limitations to deployment in biological microenvironments (clinical environments). MEF-based fluorescence detection technology with high sensitivity and selectivity will be an important key to advancing the development of portable high-sensitivity field tests that are expected to facilitate early disease diagnosis.

## Data Availability

Not applicable.
